# Effect of PI3K-p110α Inhibitor Alpelisib in the Differentiation and Effector Functions of M-CSF and GM-CSF Macrophages

**DOI:** 10.3390/ijms27104171

**Published:** 2026-05-07

**Authors:** Cristina Villa-Gómez, Verónica Bermejo, Inmaculada Márquez-Leiva, Jana Baranda, Alejandro C. Briones, Isabel Cervera, Jordi Ochando, José María Rojo, Pilar Portolés

**Affiliations:** 1 Immunology Department, National Centre for Microbiology (CNM), Carlos III Health Institute (ISCIII), 28220 Madrid, Spain; 2 Microbiology Section, Department of Pharmaceutical and Health Sciences, Faculty of Pharmacy, San Pablo-CEU University, 28668 Madrid, Spain; 3 Department of Immunology, Complutense University School of Medicine, i+12 Research Institute, 28040 Madrid, Spain; 4 Department of Oncological Sciences, Icahn School of Medicine at Mount Sinai, New York, NY 10029, USA; 5 Margarita Salas Center for Biological Research (CIB), Spanish National Research Council (CSIC), 28040 Madrid, Spain

**Keywords:** phosphatidylinositol-3-kinases, class I PI3-kinases, PI3K-p110α, monocyte, macrophage, M-CSF, GM-CSF, T lymphocyte, alpelisib (BYL719)

## Abstract

Phosphatidylinositol-3-kinases (PI3Ks) are heterodimers of catalytic and regulatory subunits that regulate cell metabolism, activation, and survival. PI3K, particularly the p110α catalytic isoform, is frequently mutated in cancer, and highly specific inhibitors such as alpelisib are currently used in oncology and in *PIK3CA*-related overgrowth disorders. Given the relevance of macrophages in anti-tumor immunity, we examined the impact of alpelisib on murine monocytes’ intracellular signaling and on in vitro differentiation, polarization, and effector functions of macrophages. Real-time qPCR (RT-qPCR) showed comparable relative expression of PI3K isoforms (p110α, p110β, p110δ, p110γ and p85) in bone marrow monocytes and in macrophages differentiated with macrophage colony-stimulating factor (M-CSF) or granulocyte-macrophage colony-stimulating factor (GM-CSF). However, alpelisib increased p110α, p110β, and p85 relative gene expression (2–3-fold) during M-CSF-dependent differentiation. Functionally, alpelisib-treated M-CSF macrophages displayed enhanced interleukin (IL)-6 and tumor necrosis factor alpha (TNF-α) secretion and reduced IL-10 production after lipopolysaccharide (LPS) plus interferon gamma (IFN-γ) or LPS stimulation. In contrast, GM-CSF macrophages differentiated with alpelisib secreted lower levels of IL-6 and TNF-α and reduced inducible nitric oxide synthase (iNOS) and arginase-1 (Arg-1) gene expression. Additionally, cytokine profiles (IL-2, IL-6, IFN-γ and IL-10) were altered when alpelisib-treated macrophages were cocultured with CD4^+^ T cells under either antigen-specific or polyclonal activation conditions, indicating that the inhibitor modifies both differentiation and subsequent effector interactions of the macrophages. Thus, alpelisib induces lasting effects on macrophage differentiation and function, with potential implications in tumor-associated macrophages that develop under M-CSF or GM-CSF-rich cancer microenvironments.

## 1. Introduction

Class I Phosphoinositide-3-Kinase (PI3K) lipid kinases are critical mediators of signals regulating cell activation, metabolism, and survival. The dysregulation of this cascade is therefore a common feature in carcinogenesis and PI3Ks play a fundamental role in the control of inflammation [1,2,3]. Distinct Class I PI3K isoforms are crucial for signaling in B cells, T cells, and innate cells such as macrophages, and are essential for maintaining adaptive immunity and self-tolerance [4,5,6,7,8,9,10].

Class I PI3Ks are heterodimeric proteins that interact through their regulatory subunits with tyrosine-phosphorylated recognition motifs located within the cytoplasmic domains of receptor tyrosine kinases (Class IA) or associated adaptor proteins, such as the Gβγ subunits of G protein-coupled receptors (Class IB) [11,12,13]. Upon cellular stimulation, Class I PI3Ks are recruited to the plasma membrane, inducing the phosphorylation of the 3-OH group of the inositol ring in phosphatidylinositol-4,5-bisphosphate (PI(4,5)P2, PIP2) to generate phosphatidylinositol-3,4,5-triphosphate (PtdIns(3,4,5)P3, PIP3). Subsequently, PIP3 recruits effector proteins like phosphoinositide-dependent kinase-1 (PDK-1), protein kinase C (PKC), Bruton’s tyrosine kinase (BTK) and protein kinase B (AKT) containing pleckstrin homology domains [14]. PI3K signaling is controlled by PI phosphatases, notably SH2 domain-containing inositol 5′-phosphatase (SHIP) and phosphatase and tensin homolog (PTEN) [15,16].

In mammals, Class IA p110α, p110β, or p110δ catalytic subunits can dimerize with different regulatory subunits (p85α, p50α, p55α, p85β, or p55γ) to form PI3Kα, PI3Kβ and PI3Kδ, respectively [11,13]. Similarly, Class IB comprises a single catalytic subunit, p110γ, associated with a regulatory subunit (p101 or p87), constituting PI3Kγ [2,12,13,17]. The abundance of these catalytic and regulatory subunits across cell types and tissues dictates their functional significance and enables their selective modulation through inhibition or deletion. While class IA catalytic subunits p110α and p110β are ubiquitously expressed in numerous cell types, p110δ and p110γ are mainly expressed in hematopoietic cells, including myeloid and lymphoid cells and B and T cells [2,4,6,17,18,19]. This suggests a selective modulation of the immune response by PI3K isoforms, and has led to complementary experimental approaches, i.e., development and characterization of transgenic mouse models with genetic modifications in various PI3K isoforms, and the generation of inhibitors specific for PI3K subunits [7].

Class I PI3Ks conduct signal propagation downstream of various cell surface receptors of leukocytes, including T cell (TCR) or B cell (BCR) antigen receptors, G protein-coupled receptors, Toll-like receptors (TLRs), and cytokine receptors, as well as T- and B-lymphocyte costimulatory molecules [4,5,6,8,18,20,21,22,23]. Indeed, T and B lymphocytes express high levels of PI3Kα and PI3Kδ, which are recruited and activated following antigen recognition and signaling through costimulatory receptors (CD19 in B cells, and CD28 or ICOS in T cells). Consequently, in lymphocytes, p110δ and p110α are essential for the development of efficient adaptive responses and act as pivotal regulators in clonal selection, differentiation and trafficking. Particularly, previous data from our laboratory showed a role of p110α in the differentiation and/or homeostasis of thymocytes, thymic regulatory T (Treg) cells, and helper T (Th) cells, or in cytokine secretion [24,25,26,27,28,29,30].

In macrophages, Class IA and IB PI3K isoforms p110β and p110γ, and to a lesser extent p110α and p110δ, participate in TLR-mediated cytokine secretion and oxidative burst, as well as in the regulation of Fc gamma receptor (FcγR) and complement receptor 3 (CR3)-mediated phagocytosis [31,32,33]. The PI3K/AKT pathway controls macrophage survival, migration, and proliferation, or their response to different metabolic signals and their activation phenotype [10,22,34,35]. PI3K activation is essential to restrict pro-inflammatory responses and promote anti-inflammatory responses in TLR-stimulated macrophages, and it is considered a negative regulator of TLR and nuclear factor kappa-light-chain-enhancer of activated B cells (NF-κB) signaling [10,31,34].

Macrophages exhibit remarkable functional plasticity, adopting a wide range of activation or polarization states in response to diverse environmental cues [35,36,37,38]. Early in vitro studies led to a simplified framework of macrophage polarization, describing two extreme activation states: classically activated, pro-inflammatory M1 macrophages and alternatively activated, anti-inflammatory M2 macrophages. Classical activation is typically induced by interferon-γ (IFN-γ), lipopolysaccharide (LPS), tumor necrosis factor-α (TNF-α), and granulocyte-macrophage colony-stimulating factor (GM-CSF), and is associated with the upregulation of inducible nitric oxide synthase (iNOS). In contrast, alternative activation is promoted by interleukins IL-4, IL-13, and IL-10, as well as immune complexes, glucocorticoids, and macrophage colony-stimulating factor (M-CSF), resulting in a highly heterogeneous population collectively referred to as M2 macrophages, which includes the M2a, M2b, M2c, and M2d subtypes [31,39,40,41,42,43]. Arginase-1 is an enzyme, a key modulator of arginine metabolism in macrophages and is commonly used as a hallmark gene of alternative activation (reviewed in [44]).

However, macrophage polarization is not a binary process but rather represents a continuous functional spectrum, with the M1/M2 paradigm describing only the extreme ends of polarization states. Accordingly, both in vitro and, more prominently, in vivo, macrophages display a broad diversity of phenotypes whose surface markers, cytokine profiles, and enzymatic activities are shaped by microenvironmental conditions, nutrient availability, and the duration and nature of stimulation, among other factors [38,44,45,46]. This pronounced heterogeneity, together with the frequent overlap of phenotypic markers, complicates macrophage classification based on rigid or strictly defined schemes. Consistent with this view, macrophages differentiated with GM-CSF or M-CSF should not be identified as M1 or M2 macrophages, respectively.

Although the p110α isoform is expressed by lymphoid and myeloid cells, sometimes at levels similar to p110δ [22,24,47], its overall impact on the immune system, and particularly in innate immune cells like monocytes and macrophages remains poorly understood.

PI3K signaling is one of the most frequently activated pathways in cancer, and *PIK3CA*, the gene encoding p110α, is a frequently mutated oncogene. Hence, there is significant interest in developing therapeutic PI3K inhibitors [1,48,49,50]. Particularly, alpelisib (BYL719) is a highly specific p110α inhibitor currently used for advanced breast tumors that has also been approved as a treatment for *PIK3CA*-related overgrowth spectrum, a group of rare, heterogeneous disorders caused by activating variants of *PIK3CA* [50,51,52,53,54].

On the other hand, LPS is the main cause of sepsis and infection-related complications in patients with cancer and other chronic diseases [55]. The involvement of PI3K isoforms in LPS-mediated cytokine induction in macrophages has often yielded controversial results, probably due to the use of non-specific isoform inhibitors or differences in the cell systems employed (i.e., monocytic tumor lines or macrophages of different origins [33,34,56,57]).

Here, we analyzed the gene expression of distinct Class I PI3K isoforms in bone marrow-derived mouse monocytes and macrophages, before and after in vitro differentiation. Furthermore, we employed alpelisib to analyze the involvement of PI3K p110α in macrophage differentiation from bone marrow monocytes treated with M-CSF or GM-CSF. We also assessed the impact of alpelisib on secretion of pro- or anti-inflammatory cytokines and iNOS/Arg-1 in response to LPS, and the modification of the cooperative function of macrophages with T lymphocytes. Additionally, our data may shed light on the potential impact of alpelisib in tumor-associated macrophages.

## 2. Results

### 2.1. Impact of Alpelisib on PI3K-Mediated Signaling in Monocytes and PI3K Isoform Expression During Monocyte-to-Macrophage Differentiation

Unlike other catalytic PI3K isoforms (i.e., p110δ or p110γ), p110α is ubiquitously expressed throughout the organism, and therefore virtually any cell type may be sensitive to the effects of alpelisib through alterations in PI3K-mediated signal transduction. Accordingly, we examined whether alpelisib modulated early activation signals induced by M-CSF or GM-CSF in purified C57BL/6J monocytes. Alpelisib inhibited the phosphorylation of signaling mediators within the PI3K pathway, including phospho-Akt, phospho-mTOR, and phospho-ERK, to different extents in M- or GM-CSF-activated monocytes (Figure S1). In the JAK/STAT pathways, alpelisib increased phospho-STAT1 and slightly decreased phospho-STAT5 phosphorylation in GM-CSF-mediated monocyte signaling (see Section 4.7 and Figure S1 for details).

**Figure 1:**
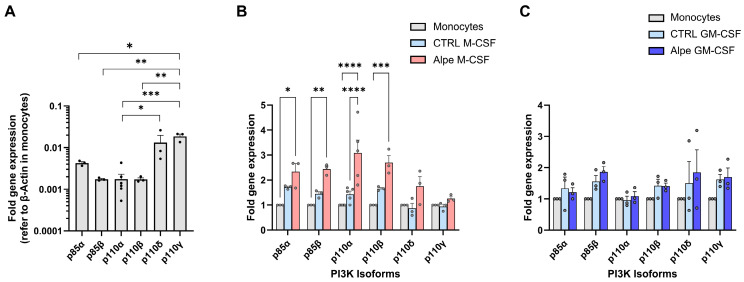
Effect of alpelisib on PI3K isoform gene expression during the differentiation of monocytes to M- and GM-CSF macrophages. Gene expression of PI3K isoforms, including regulatory (p85α and p85β) and catalytic (p110α, p110β, p110δ and p110γ) isoforms, is assessed by RT-qPCR, using β-Actin as the endogenous reference gene. (**A**) Relative gene expression of each PI3K isoform in monocytes. Statistical analysis (One-way ANOVA with multiple comparisons test) comparing the expression of the different PI3K isoforms is shown. Panel A shows the mean ± SEM of three independent experiments (5 in the case of p110α) using independent biological samples, each one analyzed in duplicate. (**B**,**C**) Fold change in gene expression in macrophages differentiated with M-CSF (**B**) or GM-CSF (**C**) relative to the expression in monocytes (set to 1). Differentiation in the presence of alpelisib (Alpe, 1 µM) is indicated by red bars (**B**) or dark blue bars (**C**). Differentiation in the presence of vehicle (0.02% DMSO, CTRL) is shown in light blue (**B**,**C**). The graphs show the data (dots) and mean ± SEM of three independent experiments, using independent biological samples, each one performed in duplicate. Each dot represents the average of duplicates in each experiment. Panel B shows p110α data from five independent experiments, each one performed in duplicate. Statistical analysis is performed using two-way ANOVA with multiple comparisons. Significant differences indicated by brackets are shown as * *p* = 0.05; ** *p* < 0.01; *** *p* < 0.001; **** *p* < 0.0001.

To analyze the effect of alpelisib in macrophage differentiation and function, we first studied potential changes in the expression of class I PI3K isoforms during M-CSF- or GM-CSF-induced differentiation of C57BL/6J monocytes into macrophages. Gene expression was analyzed by real-time quantitative PCR (RT-qPCR). Figure 1A shows the fold gene expression (2^−(∆∆Ct)^) of PI3K isoforms in purified murine bone marrow monocytes relative to the β-actin gene, as an endogenous gene control. Monocytes exhibited higher relative expression of p110γ compared with the other isoforms, except for p110δ; moreover, p110δ expression was higher than that of p110α.

In M- or GM-CSF-differentiated macrophages (Figure 1B,C), we analyzed the expression of PI3K isoforms relative to the β-actin gene in the same sample (ΔCt) and then calculated the fold gene expression (2^−ΔΔCt^) relative to the same gene in monocytes. In control M-CSF differentiated macrophages, we observed a slight, non-significant increase in the expression of PI3K regulatory (p85α and p85β), and catalytic (p110α and p110β) subunits compared with monocytes. However, differentiation in the presence of alpelisib significantly upregulated (2-3-fold) the relative expression of these four PI3K isoforms compared with monocytes. Conversely, p110δ and p110γ levels did not show significant differences in M-CSF macrophages, regardless of treatment.

In GM-CSF macrophage differentiation, no significant changes were observed in any of the evaluated PI3K isoforms compared to monocytes (Figure 1C).

### 2.2. Effect of Alpelisib on M-CSF- and GM-CSF-Macrophage Differentiation and Subsequent Polarization

To study the role of PI3K-p110α in macrophage differentiation, C57BL/6J mouse bone marrow monocytes were purified (see Section 4.3 and Figure S2A for details) and cultured for 6 days in M-CSF- or GM-CSF-supplemented media in the presence of alpelisib or vehicle (DMSO). The resulting cell cultures were characterized by flow cytometry, with murine macrophages identified by gating F4/80^+^CD11b^+^ cells (Figure S2B,C). The expression of additional cell surface markers (including CD206, Ly6c, MHC-II and CD80) was further assessed by multicolor flow cytometry in M-CSF-differentiated macrophages (M-MFs) (Figure S2B) and GM-CSF-differentiated macrophages (GM-MFs) (Figure S2C). M-MFs were characterized as F4/80^++^CD11b^++^CD206^+^Ly6c^−^CD80^+^MHC-II^++^ (Figure S2B). While M-CSF macrophages differentiated in the presence of alpelisib exhibited lower F4/80 expression (*p* = 0.038), no significant changes were observed in other markers or in cell viability, although fewer cells were recovered from alpelisib-treated cultures (Figures S2 and S3).

On the other hand, GM-MFs were characterized as F4/80^+^CD11b^+^CD206^±^Ly6c^+^CD80^++^MHC-II^++^ cells (Figure S2C), highlighting phenotypic differences between M- and GM-MF subsets. GM-MFs differentiated in the presence of alpelisib showed a significantly lower Ly6c expression (*p* = 0.02) but no changes in other surface markers or cell viability were observed (Figure S3).

To analyze the effect of alpelisib on the effector functions of macrophages, M-or GM-MFs were polarized in the presence of LPS combined with IFN-γ or IL-4, to promote macrophages with different phenotypes. Thus, pro-inflammatory (IL-6, TNF-α) or anti-inflammatory (IL-10) cytokines were measured in macrophage culture supernatants as an indication of their effector functions. To compensate for differences in cell numbers between wells of alpelisib-treated and control M-MFs cultures, cytokine quantification in culture supernatants was normalized to the mean number of cells recovered on day 6 from control (DMSO-treated) cultures by applying a correction factor (see Section 4.4).

**Figure 2:**
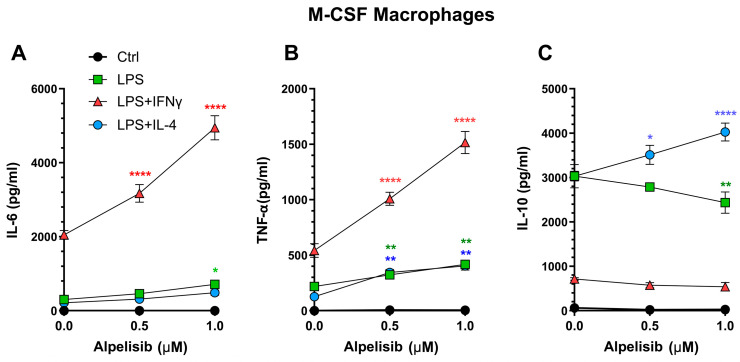
PI3K-p110α inhibition by alpelisib during M-CSF macrophage differentiation modulates cytokine secretion upon polarization. Murine bone marrow monocytes are differentiated for six days with M-CSF in the presence of increasing concentrations of alpelisib or vehicle (DMSO; 0.0 Alpelisib). On day 6, the culture supernatants are removed, and cells are polarized with the indicated stimuli. After 24 h, levels of secreted IL-6 (**A**), TNF-α (**B**), and IL-10 (**C**) are quantified by ELISA. Data represent mean ± SEM of three independent experiments with three determinations per condition. Analysis by two-way ANOVA with multiple comparisons with the control (vehicle) is performed: * *p* < 0.05; ** *p* < 0.01; **** *p* < 0.0001. Colored asterisks indicate each stimulus.

Notably, when M-MFs differentiated in the presence of alpelisib were polarized with LPS plus IFN-γ, the secretion of pro-inflammatory cytokines IL-6 and TNF-α was significantly enhanced (Figure 2A,B). In contrast, IL-10 secretion was not upregulated (Figure 2C). LPS alone, or LPS plus IL-4 induced low levels of IL-6 and TNF-α but high amounts of secreted IL-10 that were decreased (LPS) or increased (LPS+IL-4) in M-MFs differentiated in the presence of alpelisib (Figure 2C). Overall, these results suggest that M-MF differentiation in the presence of alpelisib promotes a bias towards the production of the pro-inflammatory cytokines IL-6 and TNFα when M-MFs are subsequently polarized.

**Figure 3:**
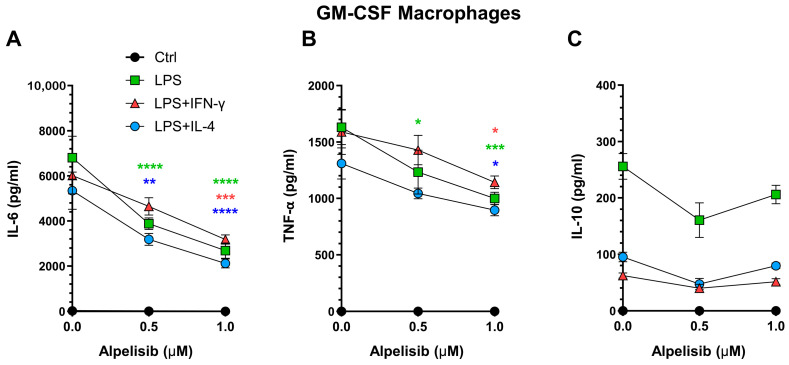
PI3K-p110α inhibition by alpelisib during GM-CSF macrophage differentiation modulates cytokine secretion upon polarization. Murine bone marrow monocytes are differentiated for six days with GM-CSF in the presence of increasing concentrations of alpelisib or vehicle (DMSO; 0.0 Alpelisib) as shown in the graphs. Then, the culture supernatants are removed, and the cells are polarized with the indicated stimuli. After 24 h, levels of secreted IL-6 (**A**), TNF-α (**B**), and IL-10 (**C**) are quantified by ELISA. Data represent mean ± SEM of three independent experiments with three determinations per condition. Analysis by two-way ANOVA with multiple comparisons to the control (DMSO) is performed * *p* < 0.05; ** *p* < 0.01; *** *p* < 0.001; **** *p* < 0.0001. Colored asterisks indicate each stimulus.

The effect of alpelisib on the differentiation of monocytes to GM-MFs was also assessed. As expected, upon polarization, GM-MFs secreted higher levels of pro-inflammatory cytokines compared with M-MFs (Figure 3A,B). However, in this case, unlike in M-MFs, alpelisib produced a clear inhibition of IL-6 and TNFα secretion induced by LPS polarization, alone or combined with IFN-γ or IL-4. In addition, GM-MFs secreted lower amounts of anti-inflammatory IL-10 compared with M-MFs, and no significant differences were observed with GM-macrophages differentiated in the presence of alpelisib (Figure 3C).

Inducible nitric oxide synthase (iNOS) and arginase-1 (Arg-1) are key enzymes involved in the regulation of macrophage functions. To assess their modulation, we analyzed the gene expression of iNOS and Arg-1 in M- and GM-MFs differentiated in the presence or absence of alpelisib for 6 days and subsequently polarized with stimuli (LPS ± IFNγ) for 24 h.

As shown in Figure S4A,B, M-MFs exhibited very low basal levels of iNOS and Arg-1 expression, similar to those observed in monocytes. These levels were markedly increased upon polarization with LPS + IFNγ. Notably, the presence of alpelisib during M-MF differentiation (6 days) did not exert a significant effect on the induction of either gene upon polarization. In polarized alpelisib-differentiated M-MFs, the magnitude of Arg-1 gene induction was higher than that observed for iNOS (Figure S4A,B).

In GM-MFs, iNOS gene expression was strongly upregulated following polarization with LPS + IFNγ (24 h). However, the presence of alpelisib during macrophage differentiation significantly inhibited the polarization-induced increase in iNOS expression (Figure S4C). Polarization of GM-MFs with LPS ± IFNγ also resulted in increased Arg-1 gene expression (Figure S4D), although to a lesser extent than that observed in M-MFs (Figure S4B). Importantly, in GM-MFs, differentiation in the presence of alpelisib markedly reduced the polarization-induced upregulation of Arg-1 expression (Figure S4D).

Overall, these results obtained using alpelisib as a PI3K-p110α inhibitor suggest that this isoform may exert a distinct regulatory role in the differentiation of M- and GM-derived macrophages and in their subsequent polarization.

To further explore the role of PI3K-P110α in the effector functions of macrophages, we analyzed the effect of alpelisib added directly during the polarization phase of M- and GM-MF induced by LPS, alone or combined with IFN-γ or IL-4 (Figure S5). Under these conditions alpelisib inhibited the IL-6 secretion by M-MFs polarized with LPS+IFN-γ or LPS alone (Figure S5A) but showed no significant effect on TNF-α or IL-10 secretion (Figure S5B,C).

When GM-MF polarization was performed in the presence of alpelisib, no statistically significant effects were found on IL-6, TNF-α or IL-10 levels (Figure S5D–F).

### 2.3. Effect of Alpelisib on APC-Independent Activation of CD4^+^ T Lymphocytes

Animal models of induced deletion or inactivation of PI3K-p110α together with in vitro assays have shown that this PI3K isoform plays a role in T cell differentiation and function [24,25,26,27,28,29,30,58]. To assess the direct effect of the PI3K-p110α inhibitor alpelisib on the secretion of cytokines by CD4^+^ T lymphocytes, we first used an activation model without antigen-presenting cells (APCs). TCR/CD3 activation of purified C57BL/6J CD4^+^ T cells was induced by plate-bound anti-CD3ε in the presence of soluble anti-CD28 antibody, plus varying concentrations of alpelisib. After 72 h of culture, the supernatants were collected and cytokines determined by ELISA.

**Figure 4:**
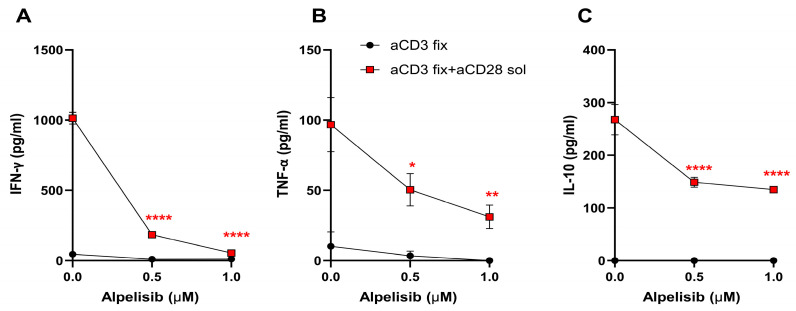
Effect of PI3K-p110α inhibitor alpelisib in the secretion of cytokines by Th CD4^+^ lymphocytes. Purified CD4^+^ T cells are stimulated with plate-bound anti-CD3 antibody (aCD3 fix) in the presence or absence of soluble anti-CD28 antibody and treated with increasing concentrations of alpelisib or vehicle (DMSO; 0.0 Alpelisib). Levels of IFN-γ (**A**), TNF-α (**B**), and IL-10 (**C**) are quantified in culture supernatants after 72 h by ELISA. Data represent mean ± SEM of two independent experiments performed in duplicate. Analysis by two-way ANOVA with multiple comparisons to the control (DMSO) is performed. * *p* < 0.05; ** *p* < 0.01; **** *p* < 0.0001. Asterisk colors correspond to the specific stimulus groups.

As shown in Figure 4, the secretion of IFN-γ, TNF-α and IL-10 by CD4^+^ cells stimulated by plate-bound anti-CD3 alone was very low and no significant difference was found in the presence of alpelisib. However, purified CD4^+^ cells activated by plate-bound anti-CD3 plus anti-CD28 antibody secreted substantial amounts of IFN-γ, TNF-α and IL-10, which were significantly inhibited by the addition of alpelisib.

### 2.4. Effect of Alpelisib in Macrophage-T Helper Cell Cooperation in an Immune Response In Vitro

The crosstalk between innate and adaptive immune cells is essential for an effective immune response. To assess the involvement of p110α in this process, we performed co-culture of T cells and differentiated macrophages acting as APC. As alpelisib had a clear effect on T cell activation (Figure 4), we performed T-MF co-culture experiments comparing macrophages that had been differentiated or not in the presence of alpelisib. Purified CD4^+^ lymphocytes from the spleen of B6 SJLxOT-II (OT-II) mice, expressing an OVA-specific transgenic TCR, were used. T cell activation by antigen-specific (peptide 323–339 of the OVA antigen, OVA_323–339_) or polyclonal stimuli (anti-CD3ε as a control) was compared, in the presence or absence of LPS as a macrophage-specific stimulus. Secretion of IL-6 (predominantly produced by macrophages), IL-2 and IFN-γ (primarily produced by T cells), and IL-10 (produced by both macrophages and T cells) was quantified after 72 h of co-culture under the indicated stimulation conditions.

**Figure 5:**
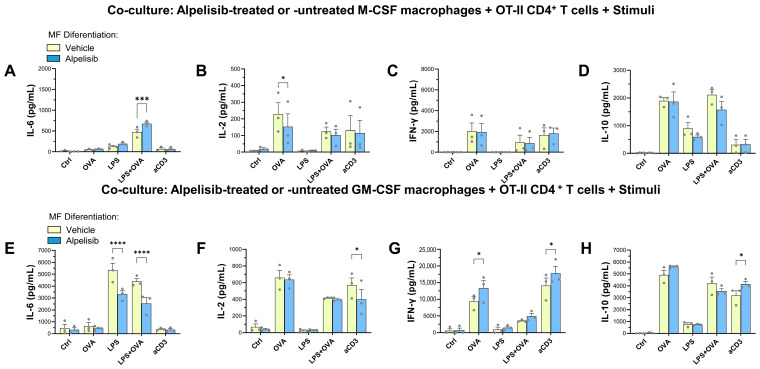
Alpelisib-induced changes in M- or GM-CSF macrophage differentiation modulate cytokine secretion during T cell responses. Purified CD4^+^ T cells from OT-II mice spleens are co-cultured for 72 h with C57BL/6J macrophages previously differentiated with M-CSF (**A**–**D**) or GM-CSF (**E**–**H**) for 6 days in the presence of 1 µM alpelisib (blue bars) or vehicle (DMSO; yellow bars). Co-cultures are stimulated with medium alone (control, Ctrl), OVA peptide 323–339 (OVA), LPS, or anti-CD3ε (aCD3) antibody. Crucially, no inhibitor is added during the co-culture phase. Cytokine levels, including IL-6 (**A**,**E**), IL-2 (**B**,**F**), IFN-γ (**C**,**G**) and IL-10 (**D**,**H**), are quantified in 72 h supernatants by ELISA. The graphs show the data (dots) and mean ± SEM of three independent experiments, using independent biological samples, each one performed in triplicate. Each dot represents the average of triplicates in each experiment. Analysis by two-way ANOVA with multiple comparisons to the respective control (without alpelisib during macrophage differentiation) is performed. * *p* < 0.05; *** *p* < 0.001; **** *p* < 0.0001.

Both C57BL/6J M- and GM-MFs differentiated in the presence of alpelisib efficiently cooperated with OT-II CD4^+^ T cells to induce an effector immune response promoting cytokine secretion in vitro. Figure 5 shows the quantification of IL-2, IL-6, IL-10 and IFN-γ in the supernatant of co-cultures under different activation conditions. In general, GM-MFs secreted or induced higher levels of cytokines than M-MFs. In the absence of T cell or macrophage-specific stimuli (OVA_323–339_, anti-CD3 Ab, or LPS), the cytokine concentrations were below the limit of detection in the assays.

IL-6 secretion was LPS-dependent in co-cultures with both M-MFs (Figure 5A) and GM-MFs (Figure 5E), while conditions containing only T cell-specific stimuli (OVA or anti-CD3) resulted in very low IL-6 levels. Notably, co-cultures with M-MFs differentiated in the presence of alpelisib exhibited increased IL-6 production (Figure 5A), whereas those containing alpelisib-treated GM-MFs showed reduced IL-6 secretion under LPS-stimulated conditions (Figure 5E). These results mirror the effects observed in cultures of M- or GM-macrophages differentiated from monocytes with or without alpelisib (Figure 2 and Figure 3).

OT-II T cells require Ag (OVA_323–339_) presentation by MHC-II^+^ cells or antibody (anti-CD3) cross-linking on FcR^+^-macrophages to become activated and secrete lymphokines such as IL-2, IFN-γ or IL-10. Under these stimulatory conditions, IL-2 secretion was slightly reduced in co-cultures containing either M- or GM-MFs differentiated in the presence of alpelisib (Figure 5B,F).

Both OVA_323–339_ and anti-CD3 induced the secretion of IFN-γ and IL-10 (Figure 5C,D,G,H). In co-cultures with M-MFs, differentiation in the presence of alpelisib did not significantly affect the secretion of these cytokines (Figure 5C,D). However, alpelisib-treated GM-MFs in the co-culture enhanced secretion of IFN-γ when OVA or anti-CD3 were present, compared with untreated macrophages (Figure 5G). Increased secretion of IL-10 in alpelisib-treated GM-MF cocultures was significant only when an anti-CD3 stimulus was present (Figure 5H).

In co-cultures with M-MFs, LPS alone induced IL-10 secretion (Figure 5D), but not IFN-γ or IL-2 (Figure 5B,C), and alpelisib treatment had no significant effect under these conditions. The combination of LPS and OVA did not produce additive effects on IL-2, IFN-γ, or IL-10 compared with OVA alone (Figure 5B–D,F–H) in the presence of either M- or GM-MFs. However, LPS+OVA did enhance IL-6 secretion relative to OVA alone when M- or GM-MFs were present (Figure 5A,E), albeit with differential outcomes depending on alpelisib treatment during macrophage differentiation.

Altogether, these findings indicate that functional inhibition of PI3K-p110α by alpelisib induces long-lasting effects during the differentiation of M- and GM-macrophages, leading to distinct functional outcomes that extend beyond macrophage development and ultimately may shape the effector T cell response.

## 3. Discussion

The fact that PI3Ks are overactivated in cancer and immune dysregulation has fostered the development of inhibitors for therapeutic purposes in cancer treatment and autoimmunity, and their impact on the immune response to infectious agents has been evaluated [7,59,60,61]. Some PI3K inhibitors have passed clinical trials and are currently in therapeutic use [59]. Mutations of class IA p110α catalytic subunits are particularly abundant in different cancers and control cell growth in solid tumors [62,63,64,65]. For these reasons, the highly specific functional inhibitor of PI3K-p110α, alpelisib (BYL719), is currently being used in combination therapy with fulvestrant in breast tumors carrying a *PIK3CA* mutation. Additional therapeutic opportunities for PI3K-p110α inhibitors beyond cancer include *PIK3CA*-related overgrowth spectrum (PROS), obesity, and metabolic syndrome [59].

PI3K-p110α is ubiquitously expressed in cells throughout the body, and its role in angiogenesis, impacting both embryonic and tumor development, has been described. Thus, part of alpelisib’s effect on tumors could be due to its impact on stromal cells, including endothelial cells, fibroblasts, and immune cells in the tumor environment [66,67,68]. Therefore, in this study, we aimed to highlight the differential effects of alpelisib on cells of myeloid or lymphoid lineages, involved in innate and adaptive immunity, particularly in in vitro models of LPS-activation, as the most common mediator of sepsis and septic shock [55], or specific-antigen response. Thus, we have shown that alpelisib-mediated inhibition of PI3K-p110α has distinct effects in the differentiation and function of two phenotypes of macrophages, namely those generated by the differentiation of murine bone marrow monocytes in M-CSF or in GM-CSF, and on T CD4^+^ lymphocytes.

The relevance of the different PI3K isoforms in cell signaling is firstly influenced by their expression level in each cell type. In addition, different catalytic isoforms show selective interactions with and activation by Ras family members [69,70]. Furthermore, PI3K catalytic and regulatory subunits show different interaction strengths, i.e., PI3K-p110α has stronger interactions with p85 regulatory subunits than other catalytic PI3K subunits like p110β [64] and p110δ [24]. All these factors influence the ability of PI3K isoforms to contribute to cell signals. Our data show p110δ and p110γ as the most abundant isoforms in monocytes, in agreement with their importance in innate cell function (reviewed in [71]) and lower levels of p110α and p110β. For comparison, p110δ and p110γ are also present and are essential to normal B and T cell development and/or function [4,30,71]. Yet, T and B lymphocytes express p110α at levels that can equal those of p110δ [4,21,24] with redundant and non-redundant roles [21,24,26,29,30].

Using alpelisib, we demonstrate that isoform p110α participates in PI3K/Akt/mTOR signaling in monocytes. Moreover, the combination of alpelisib with either M-CSF or GM-CSF induces specific alterations that subsequently manifest during macrophage polarization. Differentiation of monocytes with M-CSF or GM-CSF alone does not significantly alter the relative expression levels of genes encoding regulatory or catalytic PI3K isoforms, with the notable exception of M-CSF macrophages differentiated in the presence of alpelisib. Under these conditions, the relative gene expression levels of the regulatory p85 and catalytic p110α and p110β subunits are significantly enhanced whereas the expression levels of p110δ and p110γ do not show significant changes. A balance in PI3K isoform expression upon inhibition, genetic ablation or inactivation is not common in most systems analyzed [24,27,30,66,72]; however, it has been described in breast tumor cells treated in vitro with alpelisib [60]. Although the effects of alpelisib on PI3K transcriptional regulation require further investigation, previous reports describing the involvement of the transcription factor FOXO3a in the regulation of *PIK3CA* (p110α) transcription, as well as of other receptor tyrosine kinases such as HER3 and IGF-1R, should be considered [73,74]. Specifically, p110α expression is regulated by FOXO3a through activation of a promoter region proximal to an untranslated exon upstream of the reported transcription start site of the *PIK3CA* gene [74]. In tumors exhibiting constitutive activation of PI3K-AKT signaling, AKT inhibition leads to FOXO activation, mTORC1 inhibition, and the consequent induction of gene expression of receptor tyrosine kinases such as HER3, IGF-1R, and InsR [73]. This process results in a compensatory transcriptional upregulation of components of the PI3K pathway itself. Consistent with this model, our data show that alpelisib induces overexpression not only of p110α but also of catalytic p110β. Notably, it has been reported that the p110β isoform can partially compensate for p110α function, although downstream Akt signaling may be altered under these conditions [60].

In M-CSF differentiated macrophages, we observe that alpelisib promotes the secretion of pro-inflammatory cytokines during LPS-induction, particularly in the presence of IFN, and also during activation in antigen response. These results would agree with those obtained in genetic models of PI3K isoform depletion (PIK3r1^−/−^ mice and in p85^−/−^ [57]) in which genetic modulation of the PI3K-AKT signaling pathway decreases LPS-activation of AKT, yielding an increased expression of IL-6 and TNF-α in macrophages, indicating that the PI3K-AKT pathway inhibits LPS activation of the MAPK pathways. Interestingly, genetic disruption of p110α interaction with RAS favors differentiation of monocytes “in vivo” and “in vitro” towards a pro-inflammatory profile, and alters functions in polarized M-CSF macrophages including phagocytosis and extravasation [75,76]. This resembles our results in M-macrophages differentiated in the presence of alpelisib.

In contrast, in GM-CSF macrophages differentiated in the presence of alpelisib, LPS-induced secretion of pro-inflammatory cytokines IL-6 and TNF-α is clearly inhibited. This is in agreement with data from differentiated pro-monocytic THP-1 cells showing that loss or silencing of p110α PI3K subunits ablates LPS-induced secretion of at least some (IL-6, IL-12p40) pro-inflammatory cytokines, although this is not true for TNF-α [33]. A similar behavior is observed in M-CSF differentiated macrophages polarized in the presence of LPS plus alpelisib (Figure S5A) and as suggested by some other data on RAW264.7 cells [56]. The different effects of alpelisib on LPS-induction of IL-6 observed when the drug is present during M-CSF-monocyte differentiation (Figure 2) or after it (Figure S5), only during LPS-induced macrophage polarization, suggest that p110α may exert a modulatory role in these processes. The implication of the time of drug exposition or the functional state of the cells (monocyte or M0-macrophage) could influence the signal pathways affected and their outcomes.

The analysis of iNOS and Arg-1 expression also revealed differences in the effect of alpelisib during M- and GM-MF differentiation. Both cell types expressed both enzymes, albeit at different levels and with distinct sensitivity to alpelisib. In alpelisib-differentiated GM-MFs, a marked reduction in the LPS+IFNγ-induced upregulation of iNOS and Arg-1 was observed. The pronounced functional importance of iNOS in GM-MFs, together with the inhibition of IL-6 and TNFα cytokine secretion, suggests a reduction in the high pro-inflammatory potential of GM-MFs differentiated in the presence of alpelisib. In contrast, in M-MFs, the increase in the secretion of pro-inflammatory cytokines was not accompanied by changes in iNOS or Arg-1 expression.

One of the key physiological functions of PI3Kα at the cellular level is to translate growth factor stimulation into signals that activate anabolic processes (glucose uptake, glycolysis, nucleotide production, protein and lipid synthesis), while concomitantly inhibiting catabolic pathways, leading to the activation of AKT and mTORC1 effectors, which in turn lead to the activation of cell proliferation and survival [59]. The Akt kinase has a major role in PI3K signaling upon macrophage activation [10,34]. Interestingly, the Akt1 isoform contributes to anti-inflammatory differentiation while Akt2 favors pro-inflammatory differentiation [10,77]. Possible differences in the ability of PI3K to activate Akt isoforms may be critical determinants underlying the distinct effects of alpelisib observed in M- and GM-MFs, but the potential capability of p110α to regulate positive or negatively some macrophage signals should be considered [33]. Consequently, modulation of PI3Kα activity is expected to have broad metabolic and functional consequences, particularly in cell types whose activation state and effector functions are tightly linked to metabolic reprogramming.

In cancer cells PI3K inhibition commonly leads to a cytostatic rather than cytotoxic effect due to nutrient deprivation, as PI3K acts as a sensor for growth factors and nutrients [59,68]. In line with these previous findings, we show here that alpelisib reduces Akt and mTORC phosphorylation in monocytes stimulated by M- or GM-CSF, together with the proliferative capacity of M-CSF macrophages without significantly altering culture viability at the dose assayed. These effects of alpelisib are considered on-target, given the well-established involvement of the PI3K/AKT pathway in glucose import and in the transcriptional regulation of genes encoding glycolytic enzymes [78]. Accordingly, drug-induced inhibition of PI3K/AKT reduces glucose uptake, leading to compensatory increases in insulin secretion (reviewed in [79]). Consistent with this mechanism, hyperglycemia has been reported in 51–65% of patients receiving alpelisib in combination with endocrine therapy in clinical trials. In most cases, hyperglycemia was asymptomatic and manageable with antidiabetic medication; however, more severe cases required dose reduction or discontinuation of the drug (reviewed in [59,79].

Cell lineage may also define the importance of the effect of PI3K inhibitors. PI3K p110α and p110δ are the main class IA catalytic subunits in T cells but p110α is the isoform preferentially bound to the regulatory subunits and ICOS [24] and p110α plays a significant role in antigen activation and differentiation of T lymphocytes [26,27,30]. In agreement with these data, we show that alpelisib strongly inhibits cytokine secretion (IFN-γ, TNF-α or IL-10) induced by TCR/CD28-mediated activation of CD4^+^ T cells.

The presence of alpelisib during monocyte to macrophage differentiation has consequences in the subsequent polarization of the macrophage and in T cell-macrophage cooperation in antigen-specific immune responses. The possible impact of alpelisib on macrophage polarization in vivo should be further analyzed, particularly in the context of anti-tumor and autoimmune responses.

In conclusion, inhibition of p110α by alpelisib affects complex signaling networks that can modulate the expression of other PI3K isoforms and receptors. The ubiquitous expression of p110α may therefore lead to distinct outcomes depending on the cell type, the stage of differentiation, and the functional context within the cellular lifespan.

The possible effect of alpelisib on cellular and epigenetic reprogramming in macrophages requires further study and deserves to be investigated. Encapsulating alpelisib in nanoparticles targeted with different molecules could facilitate targeted therapy to specific cells and open new possibilities for the use of PI3K inhibitors in different pathological environments.

## 4. Materials and Methods

### 4.1. Animals

C57BL/6J mice were bred under specific opportunistic and pathogen-free (SOPF) conditions at the animal care facility of the Instituto de Salud Carlos III (Majadahonda, Madrid, Spain), from stock purchased from Charles River Laboratories (Saint-Germain-Nuelles, France) or Janvier Labs (Le Genest-Saint-Isle, France). Sex-matched, 8–12-week-old mice were used throughout the experiments.

Mice B6 SJLxOT-II (OT-II), which express a transgenic T cell receptor specific for ovalbumin, were a generous gift of Dr. D. Sancho, Centro Nacional de Investigaciones Cardiovasculares Carlos III (CNIC, Madrid, Spain) and were bred in the animal care facility of the Instituto de Salud Carlos III (Majadahonda, Madrid, Spain).

The animals were housed in racks with individually ventilated cages, under standard temperature, humidity, and airflow conditions, with a 12:12 light-dark cycle. Sterilized water and feed were provided ad libitum. All cages contained environmental enrichment material to meet the animals’ needs and reduce stress. The animal facility staff are highly trained and comply with all certification requirements for animal care and welfare. Animal euthanasia was performed by qualified personnel following humane methods in accordance with European Union regulations, ensuring conditions that minimized animal stress. The animals were used exclusively to obtain post-mortem tissue samples. No in vivo experimentation was conducted.

This research complies with the commonly accepted “3Rs”: Replacement of animals by alternatives whenever possible, Reduction in the number of animals used, and Refinement of experimental conditions and procedures to minimize harm to animals (https://www.dpi.nsw.gov.au/dpi/animals/animal-ethics-infolink/three-rs (accessed on 10 February 2026).

All experimental procedures were approved by the Ethics and Animal Welfare Committees of the Instituto de Salud Carlos III (OEBA-Majadahonda and CEIYBA) and carried out under project license PROEX 401.8/21 (Consejeria de Medio Ambiente y Ordenación del Territorio de la Comunidad de Madrid, Madrid, Spain). All the studies involving animals were conducted in accordance with local, national and European Union guidelines.

### 4.2. Antibodies, Inhibitors and Other Reagents

The antibodies used in this study and their characteristics are listed in Table S1. Recombinant human M-CSF, recombinant murine GM-CSF, and cytokines including murine recombinant IL-4 and murine recombinant IFN-γ were obtained from Peprotech (Thermo Fisher Scientific, Waltham, MA, USA). Lipopolysaccharide (LPS) from *E. coli* serotype R515 (TLR grade) was purchased from Enzo Biochem, Inc. (Farmingdale, NY, USA).

Ovalbumin peptide 323–339 (OVA_323–339_) (Cat. No. RP10610-1; GenScript Biotech Netherlands B.V., Rijswijk, the Netherlands) was used as antigen stimulus for OT-II CD4^+^ T cells.

Alpelisib (BYL719) (Cat. S2814, Selleckchem, Houston, TX, USA) was dissolved in DMSO following the manufacturer’s instructions. Aliquots of a 5 mM stock solution of alpelisib in DMSO were kept frozen (−20 °C) until use. Subsequent dilutions for the use of alpelisib in the experiments were performed in culture medium. DMSO (0.02%) in culture medium was used as vehicle control for alpelisib.

Complete culture medium (CC), Click’s medium [80] supplemented with 10% heat-inactivated fetal bovine serum (FBSi), was used for cell culture.

### 4.3. Monocyte Purification, Differentiation and Macrophage Polarization

To obtain murine monocytes, bone marrow was extracted from the femurs and tibias of C57BL/6J mice, as previously described [81]. The cell suspension was passed through 70 µm nylon cell strainers (Corning^®^, Glendale, AZ, USA) to remove debris, and red cells were lysed with ACK lysing buffer (Gibco™, Thermo Fisher Scientific). After washing, monocytes were purified by immunomagnetic selection using the Monocyte Isolation Kit (Cat. 130-100-629; Miltenyi Biotec, Bergisch Gladbach, Germany), following the manufacturer’s instructions.

For macrophage differentiation, purified monocytes were suspended in CC medium supplemented with M-CSF (30 ng/mL, Peprotech, Thermo Fisher Scientific) or GM-CSF (30 ng/mL; Peprotech, Thermo Fisher Scientific). Cells were seeded in Greiner Bio-One μClear™ P-96 tissue culture plates (advanced TC Treated, black, flat-bottom; Ref. 655986, Greiner Bio-One, Madrid, Spain) at a concentration of 5 × 10^4^/200 µL/well for M-CSF differentiation, or 4 × 10^4^/200 µL/well for GM-CSF differentiation. To study the effect of p110α inhibitor during differentiation of monocytes, alpelisib was added to a final concentration of 0.5 µM or 1 µM. DMSO (0.02%) in culture was used as vehicle control. Cultures were run in triplicate at 37 °C, with 5% CO_2_ and 95% humidity. After three days, half of the supernatant was removed and replaced with fresh warm CC medium supplemented with the corresponding growth factors (M-CSF or GM-CSF) and inhibitor or DMSO. On day 6, culture supernatants were removed, and stimuli were added to the wells in 200 µL of CC medium, for macrophage polarization. Stimuli included: LPS (10 ng/mL) alone, or in combination with IFN-γ (100 ng/mL) or IL-4 (20 ng/mL). Where indicated, the p110α inhibitor alpelisib (0.5 µM or 1 µM, final concentration) or DMSO was added only during this polarization step. After further 24 h of culture (day 7), culture supernatants were collected for cytokine analysis by ELISA.

To evaluate cell proliferation, cultures in the μClear™ P-96 plates (Greiner Bio-One) were washed once with 150 µL of warm D-PBS containing Ca^++^ and Mg^++^ (Ref. 21030-CV, Corning) and centrifuged for 5 min at 4 °C. The supernatants were discarded by quickly overturning the plate and blotting onto paper. The wells were allowed to dry in a safety laminar airflow hood and then frozen at −20 °C. Cell proliferation (based on DNA content) was quantified using the Cyquant Cell Proliferation Assay Kit (Cat. No. C7026; Invitrogen, Thermo Fisher Scientific) according to the manufacturer’s instructions.

### 4.4. Cytokine Detection

The levels of the cytokines IL-2, IL-4, IL-6, IL-10, IFN-γ, and TNF-α were determined by capture ELISA using Ready-SET-Go! Kits (eBioscience, San Diego, CA, USA) according to standard protocols.

The presence of alpelisib during monocyte differentiation with M-CSF resulted in a reduced number of cells after 6 days of culture (Figure S3C,D), although cell viability was comparable to that of control cultures in the absence of the inhibitor. To compensate for differences in cell numbers in wells from alpelisib-treated cultures, cytokine quantification in those culture supernatants was normalized to the mean number of cells recovered on day 6 in control cultures (with vehicle, DMSO) by applying a correction factor. In contrast, monocytes differentiated with GM-CSF in the presence of alpelisib did not show significant changes in cell number after six days (Figure S3C,D); therefore, in this case no correction factor was applied for the assessment of cytokines in the culture supernatants.

### 4.5. Flow Cytometry

For flow cytometric analysis, M- and GM-CSF macrophages were prepared essentially as described in Section 4.3 but cultured in 12-well Nunc™ Multidishes with UpCell™ thermosensitive surface (Ref 174900; Thermo Fisher Scientific). Monocytes were seeded at 1–1.2 × 10^6^ cells/well (M-CSF) or 0.8 × 10^6^ cells/well (GM-CSF) in 2 mL of CC medium, with 1 µM alpelisib or 0.02% DMSO where indicated. Cells were fed on day 3, and on day 6 the plates were incubated for 10 min at 4 °C to facilitate cell detachment. The recovered cells were washed twice with PBS and stained with LIVE/DEAD™ Fixable Blue Dead Cell Stain kit (Ref.: L23105; Invitrogen, Thermo Fisher Scientific), following the manufacturer’s instructions. Fc receptors were blocked with anti-CD16/CD32 antibody in cold staining buffer (1× PBS plus 2% FBSi) and incubated for 10 min at 4 °C. Subsequently, cells were spun and stained with a mix of fluorochrome-labeled primary antibodies for 30 min at 4 °C in the dark. To assess the efficiency of monocyte purification, bone marrow and post-column cells suspension were stained with labeled antibodies recognizing murine CD45, CD11b and Ly6G (see Table S1). After monocyte differentiation, macrophage phenotype was characterized using antibodies against F4/80, CD11b, CD80, CD206, Ly6C and MHC-II. After extensive washing, the cells were analyzed by flow cytometry, gating cells according to FSC/SSC. Fluorochrome-labeled antibodies against the selected surface antigens and their appropriate isotype controls are shown in Table S1. Samples were acquired using a LSR Fortessa™ X-20 (Biosciences, Franklin Lakes, NJ, USA) and analyzed using FACSDiva™ v 8.0.1 (BD Biosciences, Franklin Lakes, NJ, USA) or FlowJo v 10.0 software (Tree Star, Inc., Ashland, OR, USA). Apoptotic cells were detected using the Annexin V-FITC Apoptosis Detection Kit (Inmunostep S.L., Salamanca, Spain) following surface staining of the cells.

### 4.6. Nucleic Acid Isolation and Real-Time-Quantitative PCR

For analysis of PI3K-isoforms gene expression, purified C57BL/6J monocytes were obtained and differentiated to macrophages as described in 4.3. Nunc 12-well Multidishes with UpCell™ thermosensitive surface (Ref. 174900, Thermo Fisher Scientific) were used for culturing and gently detaching the adherent cells on day 6 as in 4.5.

For polarized-macrophages RT-qPCR, purified monocytes obtained and treated as in Section 4.3 were seeded in Nunc 48-well Multidishes with UpCell™ thermosensitive surface (Ref. 174898, Thermo Fisher Scientific), at 3 × 10^5^ cells/well (M-CSF) or 2.5 × 10^5^ cells/well (GM-CSF) in 0.8 mL of CC medium, with 1 µM alpelisib or vehicle (DMSO) as indicated. After three days, half of the supernatant was removed and replaced with fresh warm CC medium supplemented with the corresponding growth factors (M-CSF or GM-CSF) and inhibitor/vehicle. On day 6, culture supernatants were removed, and stimuli were added to the wells in 600 µL of CC medium. Stimuli included: LPS (10 ng/mL) alone, or in combination with IFN-γ (100 ng/mL). After further 24 h of culture (day 7), the plates were incubated for 10 min at 4 °C to facilitate cell detachment.

Finally, recovered cells were washed twice with sterile 1x PBS, and the pellet (0.5 × 10^6^/aliquot) was stored at −80 °C. Total RNA was extracted from monocytes or macrophages using the RNeasy Micro Kit (Ref. 74004, Qiagen, Hilden, Germany), following the manufacturer’s instructions, and quantified using NanoDrop One spectrophotometer (Thermo Scientific™).

cDNAs were synthesized from 10 to 400 ng of RNA, the same amount in each experiment, in the presence of random hexamer primers, using the SuperScript IV First-Strand Synthesis System (Ref. 18091050; Invitrogen, Carlsbad, CA, USA) and following the manufacturer’s instructions. After reverse transcription was completed, the cDNAs were quantified spectrophotometrically using NanoDrop One.

Gene expression levels were determined by RT-qPCR using a QuantStudio™ 3 Real-Time PCR System (Applied Biosystems, 96-well, 0.2 mL, ThermoFisher). For the PCR reaction, 10 µL of PowerUp™ SYBR™ Green Master Mix (Ref. A25742 Applied Biosystems, Waltham, MA, USA), cDNA (300 ng), and 0.5 µM of each primer were used. Primer sequences are listed in Table S2. Relative gene expression was calculated using the 2^−ΔΔCt^ [82] with β-actin as the endogenous control gene and monocytes as the calibrator sample.

### 4.7. Cell Signaling and Western Blot of M-CSF and GM-CSF Activated Monocyte Lysates

To assess early activation signals triggered by M-CSF and GM-CSF, freshly purified C57Bl/6J monocytes were obtained as in 4.3. After washing, cells (3 × 10^6^/point) were suspended at 5 × 10^6^/mL in culture medium containing alpelisib (1 µM final), or vehicle (DMSO) and kept at 4 °C for 30 min. Then, M-CSF (200 ng/mL) or GM-CSF (100 ng/mL) was added, as indicated, and the cells were incubated for 15 min at 37 °C. Ice-cold PBS containing 500 µM EDTA and 200 µM Na_3_VO_4_ (5 mL/sample) was added to stop activation. After centrifugation, cells were lysed for 15 min on ice with 1% Triton X-100 (1% Triton X-100 in 50 mM Tris/HCl, 150 mM NaCl, pH 7.6, 1 mM MgCl_2_, 1 mM EGTA, 1 mM Na_3_VO_4_ and protease inhibitor cocktail (Selleck Biotechnology GmbH, Köln, Germany) at 5 × 10^6^ cells/mL. Post-nuclear lysates were mixed 3V:V with 4x SDS-PAGE sample buffer and separated in 10% acrylamide SDS–PAGE. Phosphorylated proteins and appropriate loading controls were detected by immunoblot using antibodies listed in Table S3, as described in detail in [24].

### 4.8. T Cell Isolation and Activation

Single-cell suspensions were obtained from the spleen of C57BL/6J mice following euthanasia by CO_2_ inhalation. Spleens were mechanically disrupted and filtered through a 70 µm cell strainer (Corning). Erythrocytes were lysed with ACK lysing buffer (Gibco™, Thermo Fisher Scientific), and cell yield and viability were assessed by trypan blue exclusion in a Neubauer counting chamber. CD4^+^ T cells were purified by immunomagnetic isolation using the CD4^+^ T Cell Isolation Kit (Cat. 130-104-453; Miltenyi Biotec), following manufacturer’s instructions and eventually resuspended in CC medium.

For activation assays, 96-well flat-bottom tissue culture plates were pre-coated with anti-CD3ε antibody (Y-CD3-1, [83]) at 5 µg/mL in PBS. After overnight 4 °C incubation the wells were washed extensively with sterile PBS before adding CC medium. Purified CD4^+^ T cells were seeded at 2 × 10^5^ cells/well in a final volume of 200 µL/well. Where indicated, soluble anti-mouse CD28 antibody (clone 37.51, 2.5 µg/mL final concentration) was added to the wells. After 72 h of culture, 150 µL of culture supernatant was collected for cytokine quantification by ELISA.

### 4.9. Macrophage: T Cell Co-Culture

Purified C57BL/6J bone marrow monocytes were differentiated for six days in M-CSF- or GM-CSF-containing CC medium, in the presence or absence of alpelisib, as described in Section 4.3. Naïve CD4^+^ T cells from OT-II mice were isolated from spleen cell suspensions using the Naïve CD4^+^ T Cell Isolation Kit (Cat. 130-104-453; Miltenyi Biotec).

Culture supernatants were removed from the macrophage plates, and the naïve CD4^+^ T cells were added to the M- or GM-CSF macrophages, together with the corresponding stimuli. A ratio of 3:1 of CD4^+^ T cell:macrophage was kept in the experiments considering the number of monocytes seeded in day 0 in M-CSF- or GM-CSF-containing cultures (5 × 10^4^/well and 4 × 10^4^/well, respectively). Antigen peptide OVA_323–339_ (1 µg/mL), LPS (10 ng/mL) or anti-CD3 (Y-CD3-1, 2.5 µg/mL) were used as stimuli in the co-cultures.

After 3 days, the co-culture supernatants were collected to quantify cytokines by ELISA.

### 4.10. Statistical Analyses

Data were analyzed with GraphPad Prism 10.6.1 software (GraphPad Prism Software Inc., La Jolla, CA, USA), and they are shown as the mean ± standard error of the mean (SEM). Pairwise comparisons were made using an unpaired, two-tailed Student’s *t*-test. One- or two-way ANOVA was used when multiple comparisons were necessary, as indicated in the figure legends. Significant differences between data are indicated by asterisks (* *p* < 0.05, ** *p* < 0.01, *** *p* < 0.005, and **** *p* < 0.001). All statistical calculations refer to the control group, or as indicated by brackets.

## Data Availability

The original contributions presented in this study are included in the article/Supplementary Materials. Further inquiries can be directed to the corresponding authors.

## Supplementary Materials

The following supporting information can be downloaded at: https://www.mdpi.com/article/10.3390/ijms27104171/s1. References [84,85,86,87,88,89,90,91,92] are cited in Supplementary Materials.

## Abbreviations

The following abbreviations are used in this manuscript:

APCs	Antigen-presenting cells
Arg-1	Arginase-1
BCR	B cell receptor
GM-CSF	Granulocyte-macrophage colony-stimulating factor
GM-MFs	GM-CSF-differentiated macrophages
HRP	Horseradish peroxidase
IL	Interleukin
IFN-γ	Interferon gamma
iNOS	Inducible nitric oxide synthase
LPS	Lipopolysaccharide
M-CSF	Macrophage colony-stimulating factor
M-MFs	M-CSF-differentiated macrophages
OT-II	B6 SJLxOT-II mice
OVA	Ovalbumin
PIs	Phosphoinositides
RT-qPCR	Real-time quantitative polymerase chain reaction
TCR	T cell receptor
Th	T helper cell
TLRs	Toll-like receptors
TNF-α	Tumor necrosis factor alpha
Treg	T regulatory cell
