# Supplementary material for: Effect of PI3K-p110α Inhibitor Alpelisib in the Differentiation and Effector Functions of M-CSF and GM-CSF Macrophages
Source: Int J Mol Sci. 2026 May 7;27(10):4171. doi: 10.3390/ijms27104171 (PMC13207794; doi:10.3390/ijms27104171)
Supplement: Supplementary file 1 [file ijms-27-04171-s001.zip › ijms-4173070-supplementary.pdf]

Supplementary Figures and Tables for

Effect of PI3K-p110α inhibitor Alpelisib in the differentiation and effector functions of M-CSF and GM-CSF macrophages, by Villa-Gómez et al.

Effect of alpelisib on early signaling of monocytes activated with M-CSF and GM-CSF (Figure S1).

To assess whether alpelisib modifies activation signals induced by M-CSF and GM-CSF, freshly isolated bone marrow monocytes were activated with M-CSF or GM-CSF in the presence of 1 μM alpelisib or vehicle (0.02% DMSO). Then, early activation of downstream pathways was determined by western blot as phosphorylation of different proteins in cell lysates (see section 4.7).

As shown in Figure S1, alpelisib inhibited PI3K-dependent Akt phosphorylation in M-CSF- or GM-CSF-activated monocytes, being M-CSF signaling weaker than that of GM-CSF.

M-CSF- or GM-CSF-induce MAPK-dependent ERK phosphorylation that can be modulated by PI3K (84-91). ERK activation induced by M-CSF was weaker than that of GM-CSF and both were inhibited by alpelisib. Similar results were observed in the downstream target of PI3K mTOR.

Activation of JAK/STAT pathways was determined as phosphorylation of STAT1 and STAT5. STAT1 can be activated by GM-CSF (84,85). GM-CSF-induced STAT1 phosphorylation was slightly upregulated in the presence of alpelisib, despite the control of STAT1 activation by PI3K/Akt in certain systems (91).

Last, GM-CSF induced STAT5 phosphorylation was slightly modulated by alpelisib, in agreement with data from human monocytes indicating that this is largely independent of PI3K (92).

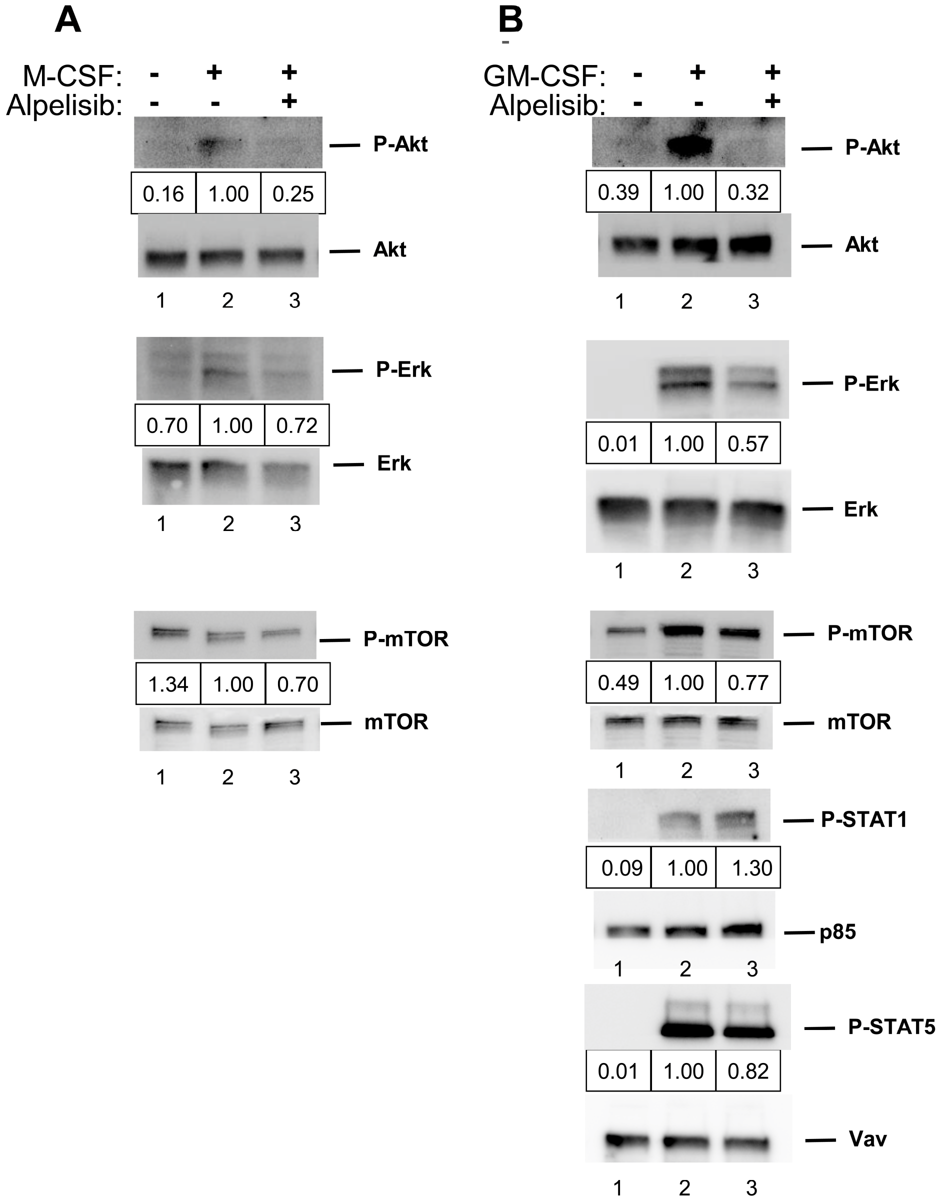

Figure S1. Effect of alpelisib on early signaling of monocytes activated with M-CSF and GM-CSF. Signaling in isolated mouse bone marrow monocytes was determined by western blot of cell lysates after 15 min of incubation with M-CSF (A) or GM-CSF (B) with 1 μM alpelisib or 0.02% DMSO. Activation was assessed as phosphorylation of AKT, ERK1/2, mTOR, STAT1, or STAT5, as indicated. Optical density of phosphorylated proteins normalized to the indicated proteins is shown inserted in rectangles.

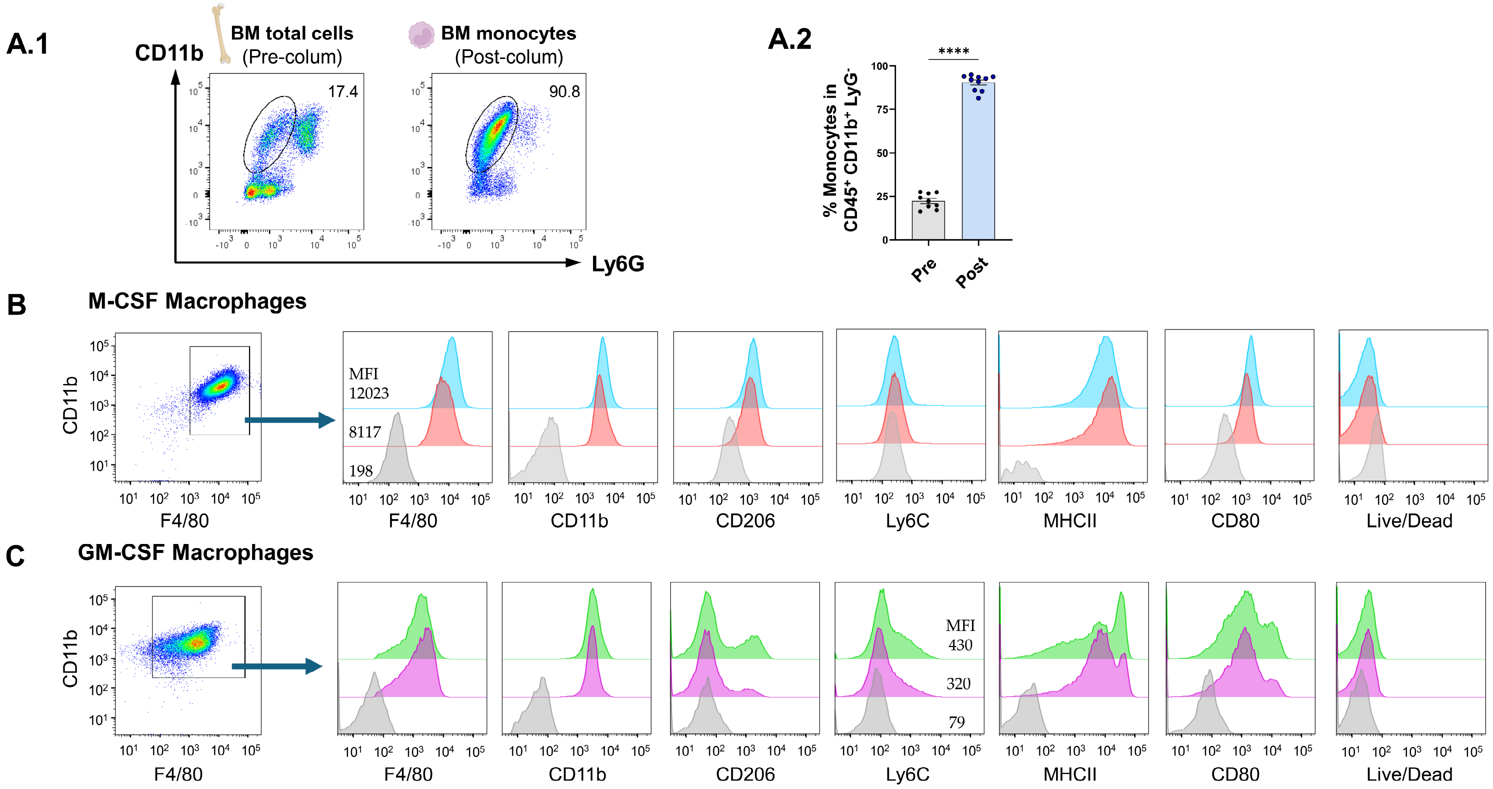

Figure S2. Flow cytometry analysis of monocyte purity and macrophage differentiation.

Bone marrow (BM) cells were obtained from the femurs and tibias of C57Bl/6J mice and processed as described in the Methods section. Antibodies used for cytometry analysis are shown in Table 1.

A. Representative flow cytometry plots showing the frequency of CD11b$^+$Ly6G$^-$ monocytes within the live, single, CD45$^+$ bone marrow cell population, after the exclusion of doublets (FSC-H/FSC-A and SSC-W/FSC-H). The analysis compares total bone marrow cells before (Pre-column, A.1, left panel) and after magnetic enrichment (Miltenyi Biotec) (Post-column, A.1, right panel) on day 0. Numbers indicate the percentage of the gated CD11b$^+$Ly6G$^-$ monocyte population. A.2. Quantification of monocyte purity, showing the percentage of CD11b$^+$Ly6G$^-$ monocytes before (22.3 ± 1.5) and after isolation (90.4 ± 1.4). The graph displays data from independent experiments for both pre-isolation (n=9) and post-isolation (n=10). Data are presented as mean ± SEM, and statistical significance was determined using an unpaired t-test; ****$p < 0.0001$.

B. Phenotypic characterization of M-CSF-differentiated macrophages. C57BL/6J monocytes were cultured in M-CSF-supplemented medium in the presence of vehicle (DMSO) (blue histograms) or 1 μ M alpelisib (red histograms) for 6 days. Macrophages were identified as F4/80$^+$CD11b$^+$ cells. Histograms show the expression of characteristic surface markers: F4/80, CD11b, CD206, Ly6C, MHC-II, and CD80. Isotype controls are shown in grey. Right panel: Viability assessment using LIVE/DEAD™ Fixable Blue staining. Statistical analysis: Comparison of the mean of fluorescence intensity (MFI) ratios (alpelisib MFI vs. control MFI) revealed a significant reduction in surface F4/80 expression in alpelisib-treated M-CSF macrophages (p= 0.038, unpaired Student's t-test, n= 4 independent experiments). No significant differences were observed for other markers.

C. Phenotypic characterization of GM-CSF-differentiated macrophages. Monocytes were cultured in GM-CSF-supplemented medium in the presence of vehicle (DMSO) (green histograms) or 1 μ M alpelisib (purple histograms) for 6 days. Isotype controls are shown in grey. The cytometry strategy was similar to that described in B. Statistical analysis: Notably, Ly6C expression was significantly diminished in alpelisib-treated GM-CSF macrophages compared to controls (p= 0.02, unpaired Student's t-test, n = 4 independent experiments).

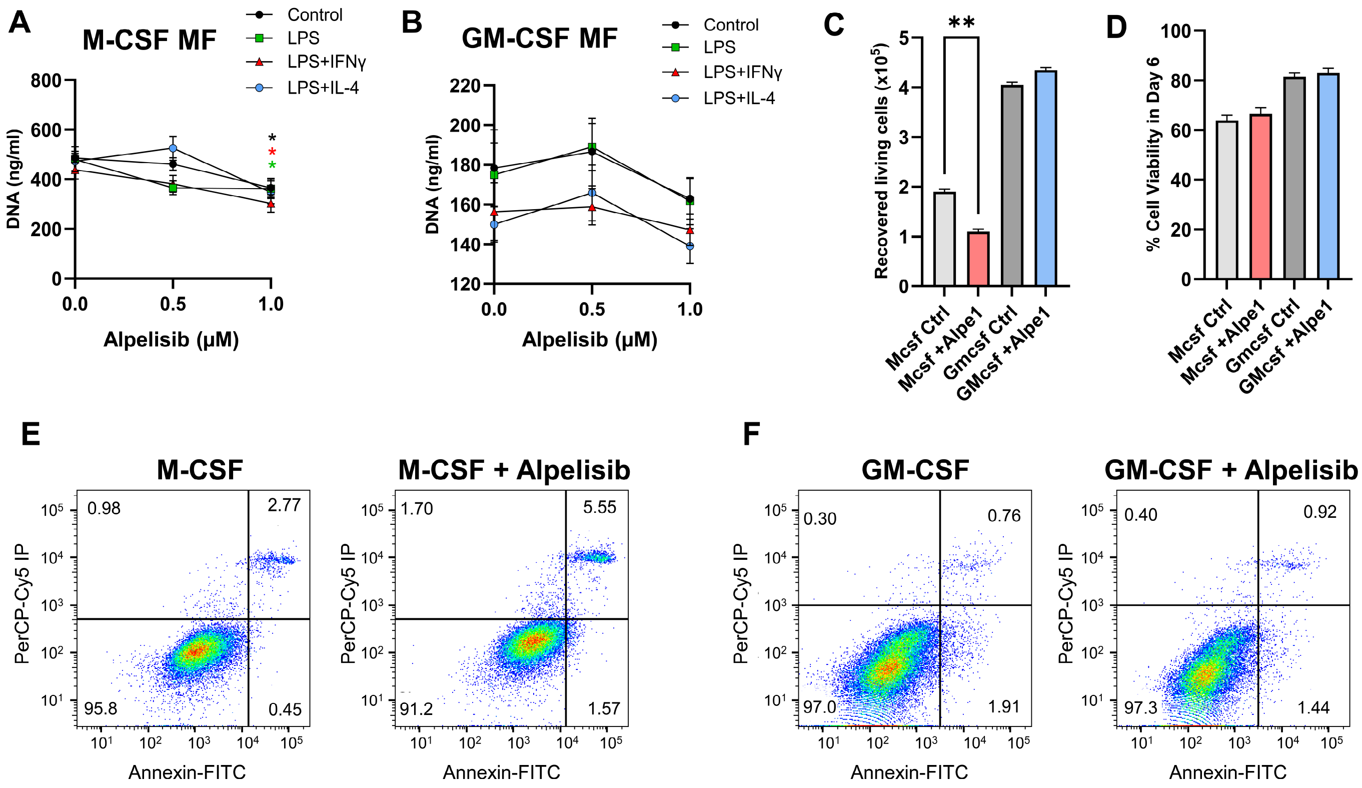

Figure S3: Effect of alpelisib in proliferation, viability and apoptosis in M- and GM-CSF macrophages.

A, B. Proliferation. Cell proliferation was assessed by quantifying DNA content using the CyQUANT™ kit (see Section 4.3). Monocytes were differentiated for 6 days with M-CSF (**A**) or GM-CSF (**B**) in the presence of vehicle (DMSO) or increasing concentrations of alpelisib, and subsequently polarized (24 h) with the indicated stimuli (LPS, LPS+IFN-γ, or LPS+IL-4). Graphs show the mean ± SEM of DNA content (ng/mL) from three independent experiments performed in triplicate. A decrease in DNA content was observed in M-CSF macrophages differentiated with 1 μM alpelisib (**A**), whereas no significant effect was detected in GM-CSF macrophages (**B**). Analysis by two-way ANOVA with multiple comparisons to the control (vehicle, DMSO) was performed. * p<0.05. Colored asterisks indicate each stimulus.

C, D. Cell recovery and viability. Monocytes were differentiated for 6 days in 12-well UpCell™ thermosensitive plates (Ref 174900; Thermo Fisher Scientific, Waltham, MA, USA) to facilitate detachment. Cells were harvested and counted using trypan blue exclusion. (**C**) Total number of recovered cells. A significant decrease in cell recovery was observed in M-CSF macrophages treated with 1 μM alpelisib compared with controls (vehicle, DMSO). (**D**) Percentage of cell viability. Analysis by unpaired T-test was performed: ** p<0.01. No significant differences in viability were found between treated and untreated groups, suggesting that the reduction in cell recovery is due to impaired proliferation rather than cytotoxicity.

E, F. Apoptosis. Apoptosis rates were evaluated by flow cytometry using Annexin V-FITC staining after surface staining of the cells (see section 4.5). Low levels of apoptosis were detected in both M-CSF (**E**) and GM-CSF (**F**) macrophages, with no significant differences observed in the presence of 1 μM alpelisib.

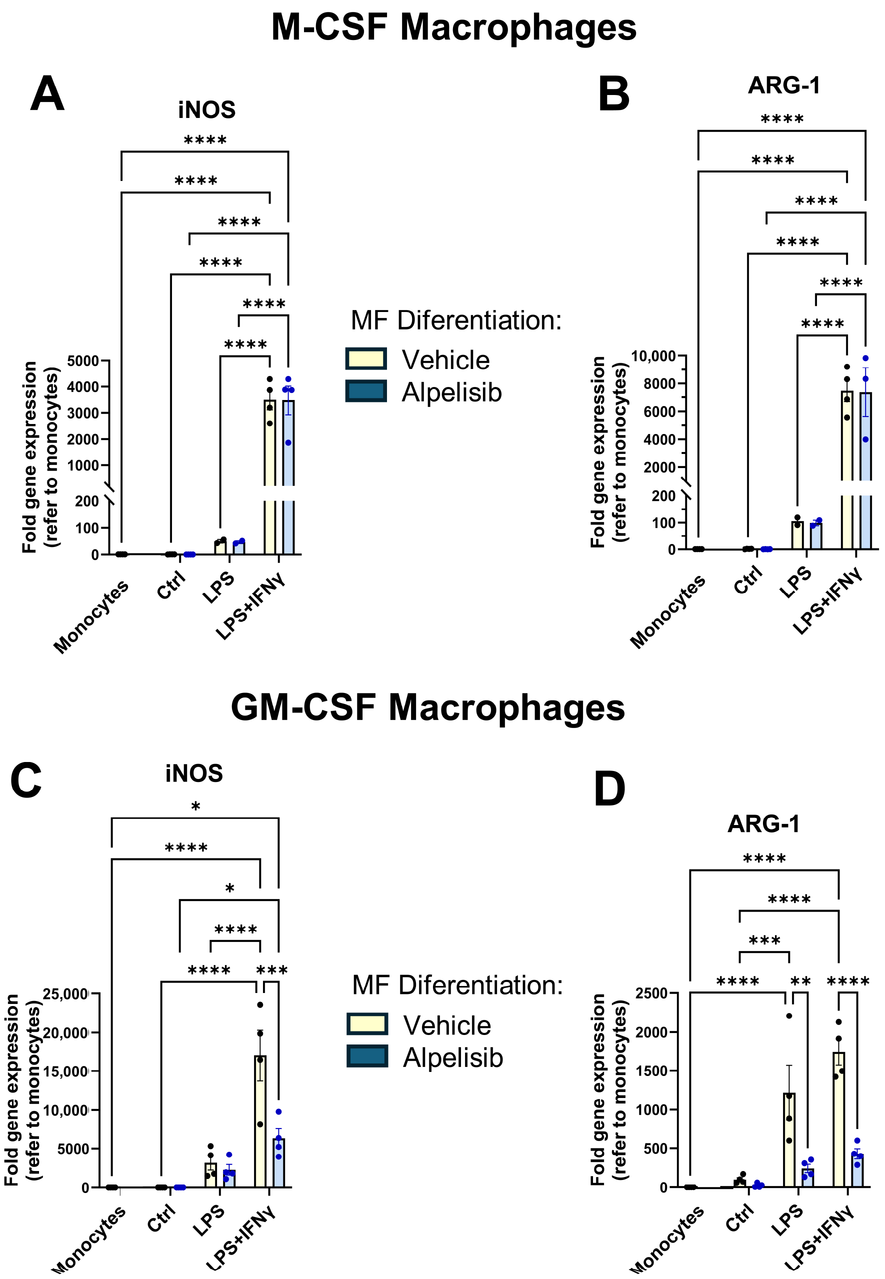

Figure S4. Gene expression of iNOS and Arg-1 in polarized M- and GM-CSF macrophages that were differentiated in the presence of alpelisib.

Fold gene expression of iNOS (A,C) and Arg-1 (B,D) in macrophages differentiated in M-CSF (A,B) or in GM-CSF (C,D) in the presence of 1μM alpelisb (blue) or vehicle (DMSO, yellow). Purified C57Bl/6J monocytes (see section 4.3) were differentiated for 6 days in the presence of 1μM alpelisib or vehicle (DMSO) and subsequently polarized (24 h) with LPS±IFNγ or culture medium (Ctrl). Then, cells were harvested, washed and the pellets were frozen at -80 ºC. Gene expression of iNOS and Arg-1 was assessed by RT-qPCR (see section 4.6), using β-Actin as housekeeping gene to calculate ΔCt. Monocytes were used as calibrator sample for ΔΔCt and fold change gene expression ($2^{-\Delta\Delta Ct}$) of iNOS and Arg-1. The graphs show the data and mean ± SEM of independent biological samples. Statistical analysis was performed using Two-way ANOVA with multiple comparisons. Significant differences indicated by brackets are shown as *p<0.5; **p<0.01; *** p<0.001; ****p<0.0001.

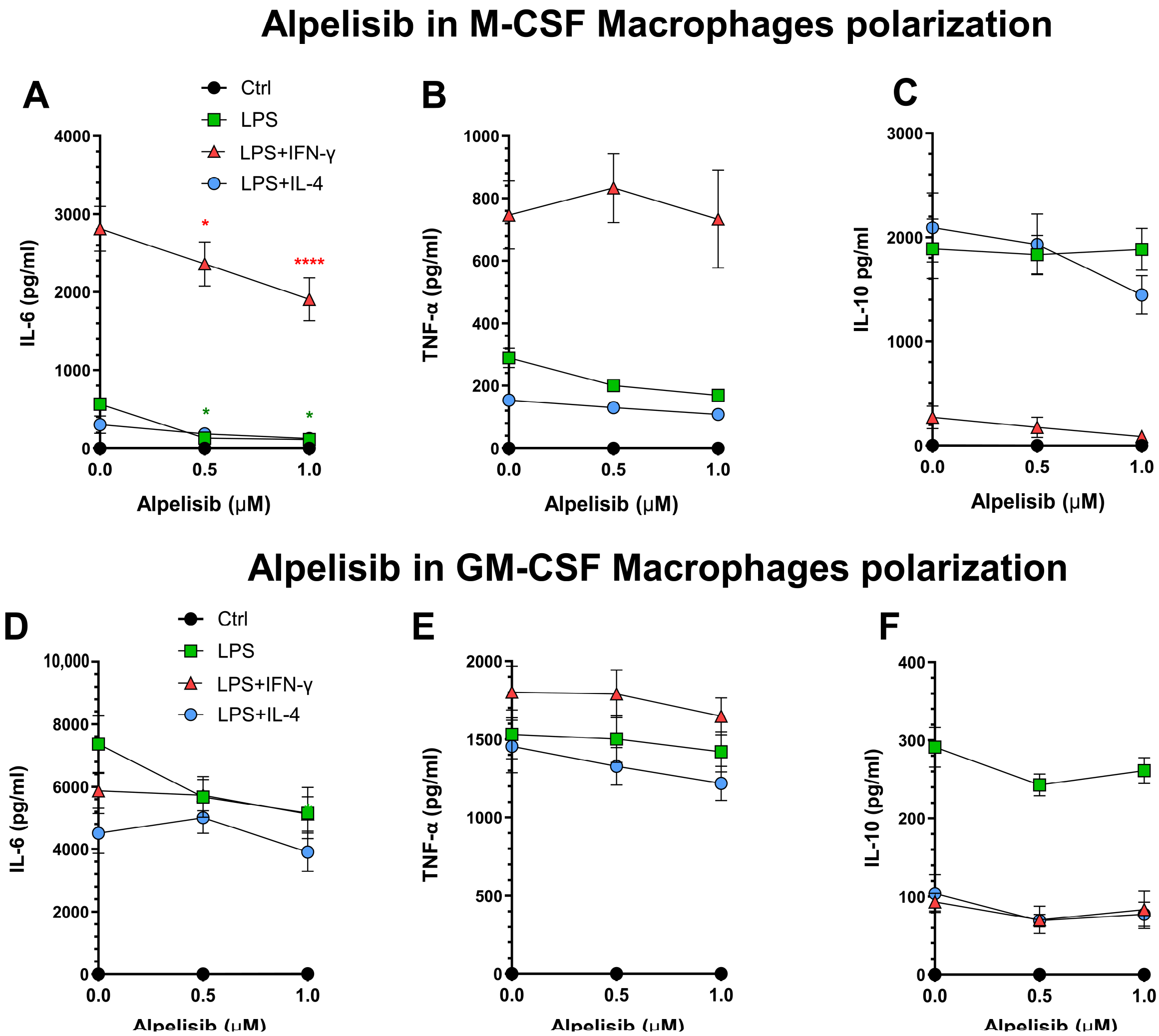

Figure S5: Effect of alpelisib in M- and GM-CSF macrophages polarization.

Monocytes were differentiated to macrophages in the presence of M-CSF (A-C) or GM-CSF (D-F) for 6 days following the methods described in Section 4.3. Then, the supernatants were removed from the wells, and different stimuli (LPS with or without IFN-γ or IL-4) were added to the wells in 200μL of CC medium. Where indicated, alpelisib (0.5 μM or 1 μM final concentration) was added to the CC medium during the polarization step only. After further 24 h of culture (day 7), culture supernatants were collected and cytokine content (IL-6, TNF-α and IL-10) was analyzed by ELISA.

Table S1. Antibodies used in flow cytometry and cell activation.

Ag specificity	Clone	Label	Species	Isotype	Manufacturer
CD3ε	Y-CD3-1	None	Rat	IgG2b	In-house production (83)
CD11b	M1/70	FITC/eF450	Rat	IgG2b	eBioscience
CD16/CD32	93	None	Rat	IgG2a	eBioscience
CD28	37.51	None	Hamster	IgG	Invitrogen
CD45	30.F11	PE / APC-eFluor	Rat	IgG2b	eBioscience
CD80	16-10A1	BV711	Hamster	IgG	BD Pharmingen
CD206	MR6F3	FITC	Rat	IgG2a	Invitrogen
Ly6C	AL-21	PE	Rat	IgM	BD Pharmingen
Ly6G	1A8	PE-Cy7	Rat	IgG2a	BD Pharmingen
F4/80	BM8	APC	Rat	IgG2a	BioLegend
MHC-II	M5/114	BV786	Rat	IgG2b	BioLegend
Isotype	eBio299Arm	BV711	Hamster	IgG	eBioscience
Isotype	RTK2758	FITC / APC	Rat	IgG2a	BioLegend
Isotype	eB149/10H5	FITC / PE	Rat	IgG2b	eBioscience
Isotype	RTK2118	PE	Rat	IgM	BioLegend

Table S2. Oligonucleotides used for RT-qPCR in this study

Name	Murine gene sequence (5´ to 3´)
β-Actin-F	TGTTACCAACTGGGACGACA
β-Actin-R	GGGGTGTTGAAGGTCTCA
p85α-F	GAATGTTCGACTCTATACAGAACACAA
p85α-R	CATCTCCAAGTCCACTGACG
p85β-F	CCCTTGGATGGATCTTCTGA
p85β-R	GGGTCAGGTGGGGAGAAC
p110α-F	GACAAGAACAAGGGCGAGAT
p110α-R	CAGTACCCAGCGCAGGAC
p110β-F	AGAAGCTGGCTTGGACCTG
p110β-R	CAGAGCGATCTCCTGTTGCT
p110γ-F	TTCTCGTGTGTCCACCATGT
p110γ-R	CCTGGGCATCTCAGTGGTAT
p110δ-F	AGCTGCTCCAAAGATATCCAGT
p110δ-R	TGCTTTAGCGCCTCTTCCT
iNOS-F	CCTGCTTTGTGCGAAGTGTC
iNOS-R	CCCTTTGTGCTGGGAGTCAT
Arg-1-F	CTCCAAGCCAAAGTCCTTAGAG
Arg-1-R	AGGAGCTGTCATTAGGGACATC
Manufacturer: Merck Life Science, Darmstadt, Germany.	

Table S3. Antibodies used for western blot.

Specificity (mouse)	Label	Clone	Species	Manufacturer
AKT1/PKBα	-	Polyclonal, Ref. 07-416	Rabbit	UpState
AKT1/PKBα- Phospho Ser473	-	D9E mAb	Rabbit	Cell Signalling Technology
MAP Kinase2 (Erk2)	-	Polyclonal, Ref. sc-154	Rabbit	Santa Cruz Biotechnology
MAP Kinase Erk 1/2- Phospho p44/42 (Thr202/Tyr204)	-	137F5 mAb	Rabbit	Cell Signalling Technology
mTOR-Phospho Ser2448	-	D9C2 mAb	Rabbit	Cell Signalling Technology
mTOR	-	Polyclonal, Ref. PA5-34663	Rabbit	Invitrogen
p85 PI3K	-	Polyclonal, Ref. 06-195	Rabbit	UpState
STAT1-Phospho Tyr701	-	D4A7 mAb	Rabbit	Cell Signalling Technology
STAT5-Phospho Tyr694	-	D47E7 mAb	Rabbit	Cell Signalling Technology
Rabbit IgG	HRP	Polyclonal, Ref. A0545	Goat	Sigma-Aldrich
Vav (mouse oncoVav$_{738-845}$-GST)	-	Polyclonal, affinity purified	Rabbit	In house
